# Gender Disparities in Japanese Interventional Cardiology: Outcomes of Female Operators in Percutaneous Coronary Interventions

**DOI:** 10.1016/j.jacasi.2024.07.005

**Published:** 2024-08-20

**Authors:** Saeko Takahashi, Kyohei Yamaji, Shun Kohsaka, Kyoko Hayashida, Junko Sato, Reiko Tsukahara, Junko Honye, Tetsuya Amano, Ken Kozuma, J-PCI Registry Investigators

**Affiliations:** a Department of Cardiology, Shonan Oiso Hospital, Oiso, Japan; b Department of Cardiology, Shonan Kamakura General Hospital, Kamakura, Japan; c Department of Cardiology, Kyoto University, Kyoto, Japan; d Department of Cardiology, Keio University School of Medicine, Tokyo, Japan; e Administration Department, Japanese Association of Cardiovascular Intervention and Therapeutics, Tokyo, Japan; f Department of Cardiology, Tokyo General Hospital, Tokyo, Japan; g Department of Cardiology, Kikuna Memorial Hospital, Yokohama, Japan; h Department of Cardiology, Aichi Medical University, Nagakute, Japan; i Department of Cardiology, Teikyo University Hospital, Tokyo, Japan

**Keywords:** female interventionalists, gender disparity, gender diversity, operators’ gender, percutaneous coronary intervention

## Abstract

**Background:**

Gender disparity remains a significant global concern in interventional cardiology, and there is a lack of extensive research examining the outcomes of percutaneous coronary interventions (PCIs) performed by female interventionalists.

**Objectives:**

The aim of this study was to examine the practice patterns and outcomes of PCIs conducted by female interventionalists in Japan.

**Methods:**

This retrospective observational study analyzed data from the Japanese Percutaneous Coronary Intervention registry between January 2019 and December 2021. The primary endpoint was in-hospital mortality and the secondary endpoint was the success rate of PCIs.

**Results:**

A total of 447 female operators (7.3% of all operators) performed 35,211 PCIs (5.3%) during the study period. Female doctors treated a higher percentage of patients with ST-segment elevation myocardial infarction compared with their male counterparts (20.2% vs 17.7%; *P* = 0.001), whereas male doctors were more likely to perform PCIs for left main disease (4.9% vs 5.9%; *P* < 0.001) and lesions treated with rotational atherectomy (3.5% vs 4.9%; *P* < 0.001). The success rate of PCIs was higher for female interventionalists (97.8% vs 97.2%; *P* < 0.001). After conducting a risk-adjusted analysis, we found no significant difference in in-hospital mortality (adjusted OR: 0.896; 95% CI: 0.78-1.03; *P* = 0.12), or procedural complications associated with the operator's gender.

**Conclusions:**

Overall, female operators exhibited outcomes similar to their male counterparts in terms of adjusted procedural outcomes, and higher crude success rate in certain subgroups. These findings emphasize gender disparities and stress the need to increase gender diversity in interventional cardiology.

Globally, women account for more than 50% of medical school graduates.^1^^,^^2^ In Organization for Economic Co-operation and Development (OECD) countries, nearly half of all doctors are women.^3^ However, Japan ranks last among OECD nations, with only 21% being women.^3^, ^4^, ^5^ The proportion of female physicians specializing in cardiology is even lower, reported to be only 2.2% in 2020,^6^ leading to limited gender diversity in interventional cardiology in the country. Reports from the United States, Australia, and Europe also indicate substantially fewer female interventional cardiologists than male.^7^, ^8^, ^9^, ^10^, ^11^, ^12^ However, little is known about percutaneous coronary intervention (PCI) practice patterns and outcomes with regard to the gender of the interventionalists.

In recent years, there has been growing attention to outcomes of medical treatment in internal medicine or surgical procedures performed by female doctors.^4^^,^^5^^,^^13^, ^14^, ^15^ Studies have shown that female doctors who care for older hospitalized patients achieve better results,^13^ and there is evidence that gender discordance between surgeons and patients negatively impacts outcomes following common operations.^14^ In the field of interventional cardiology, the relatively low number of female doctors has resulted in limited information regarding associations between gender and practice patterns, as well as outcomes, of PCI.^7^, ^8^, ^9^

This study aims to assess the representation of women performing PCI in Japan, examine the procedural volumes and case-mix of PCI procedures performed by women, and compare PCI outcomes between male and female interventionalists.

## Methods

The J-PCI (Japanese Percutaneous Coronary Intervention) registry is a prospective multicenter registry endorsed by the Japanese Association of Cardiovascular Intervention and Therapeutics (CVIT) and provides data on the performance of PCIs at a national level in Japan.^16^, ^17^, ^18^ The study protocol of the J-PCI registry was approved by a third-party ethics committee at Osaka University, and it complies with the principles contained within the Declaration of Helsinki. Because of the retrospective and observational nature of this study, written informed consent for patients was waived. Opt-out consent was offered by e-mail to all members of CVIT. Strengthening the Reporting of Observational Studies in Epidemiology (STROBE) guidelines^19^ and the Sex and Gender Equity in Research (SAGER) were observed.^20^

We included consecutive patients who underwent PCI between January 1, 2019, and December 31, 2021, using data from the J-PCI registry. Cardiac catheterization procedures are performed in publicly and privately funded hospitals in Japan. Registration in the J-PCI registry is mandatory for board certification and renewal under both systems and data completion is high. The registry database includes data from approximately 240,000 PCI cases annually, which are performed in more than 1,100 hospitals, representing more than 90% of PCI centers in Japan. Each hospital has a data manager responsible for the collection and entry of PCI data into the online database. The accuracy of submitted data is validated by a data audit (20 sites per year) performed by the members of the CVIT Registry Subcommittee, and a meeting of data managers is held annually for quality control.

### Definitions and clinical outcomes

The full definitions of these J-PCI registry variables are available in the previously published manuscript.^16^ ST-segment elevation myocardial infarction (STEMI) was defined as acute myocardial infarction with ST-segment elevation on at least 2 contiguous leads, new left bundle branch block, or posterior myocardial infarction on a 12-lead electrocardiogram, along with elevated cardiac biomarkers that are 2-fold higher than the normal values or elevated troponin levels (≥99th percentile), respectively. The definition of cardiogenic shock was a sustained episode of systolic blood pressure <80 mm Hg and a cardiac index <1.8 L/min/m^2^ caused by cardiac dysfunction. The outcomes analyzed in this cross-sectional study included the success of PCI, in-hospital all-cause mortality, procedure-related complications, and bleeding complications.

The primary endpoint was in-hospital mortality, defined as death from any cause before discharge following PCI. The J-PCI registry collects data on in-hospital mortality and complications separately for each patient, which allowed avoidance of double-counting events. The secondary endpoint was a successful PCI defined as achieving TIMI flow grade 3 with residual stenosis <25% in PCI procedures. Procedure-related complications included periprocedural myocardial infarction, cardiac tamponade, cardiogenic shock during and after PCI, emergent surgery, bleeding, and other complications during the hospitalization. Bleeding complications were defined as bleeding during or after PCI requiring blood transfusion, including access and nonaccess site bleeding. Periprocedural myocardial infarction was defined as a rise of cardiac troponin more than 5 times the cutoff threshold.

### Statistical analysis

Continuous variables are reported as mean ± SD and categorical variables are presented as frequency and percentage. We conducted a comparative analysis of the clinical characteristics of patients and procedures of PCIs performed by male and female operators. We constructed linear mixed regression models or logistic mixed regression models to assess the differences between male and female operators with respect to patients’ baseline clinical characteristics, angiographic data, procedural data, and in-hospital complications. The models included operators as random intercepts to account for clusters per operator. In addition, multivariable models were constructed to evaluate the association between female operators and in-hospital mortality, success of the PCIs, and other complications following PCIs.

The following were included into the models as explanatory variables: age, sex, hypertension, diabetes, hyperlipidemia, smoking, renal insufficiency, hemodialysis, chronic obstructive pulmonary disease, peripheral artery disease, acute heart failure, cardiogenic shock, cardiac arrest, chronic kidney failure including dialysis, extent of coronary artery disease, access site, the use of debulking device, and PCI performed on weekends. A *P* value of <0.05 was considered statistically significant. Statistical analyses were performed using R version 4.4.0 (R Foundation for Statistical Computing).

### Results

**Figure 1:**
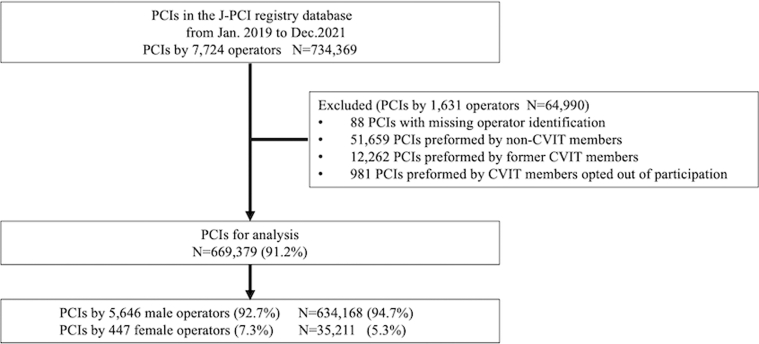
PCI Inclusion Flow Chart A total of 734,369 PCIs performed by 7,724 operators from the J-PCI registry from January 2019 to December 2021 were initially included. After excluding 64,990 PCIs performed by 1,631 operators, the final analysis included 669,379 PCIs performed by 5,646 male operators and 447 female operators. CVIT = cardiovascular intervention and therapeutics; J-PCI registry = Japanese Percutaneous Coronary Intervention registry; PCI = percutaneous coronary intervention.

Our study population consisted of 734,369 PCIs performed by 7,724 interventionalists in Japan between January 2019 and December 2021. Among them, 64,990 PCIs were excluded because of missing operator identification or being performed by non-CVIT members or by operators who opted out of participation. A total of 669,379 PCIs (91.2%) were available for analysis (Figure 1). During this study period, 447 interventionalists (7.3%) were women, and they performed 35,211 (5.3%) cases. The median age of female operators was 35 years, whereas that of men was 40 years. Over the 3-year study period, the median number of procedures performed by women per 3 years was 57 (with an IQR: 23-108), which was significantly lower than the median number of procedures performed by men (median: 92; IQR: 42-153; *P* < 0.001).

**Figure 2:**
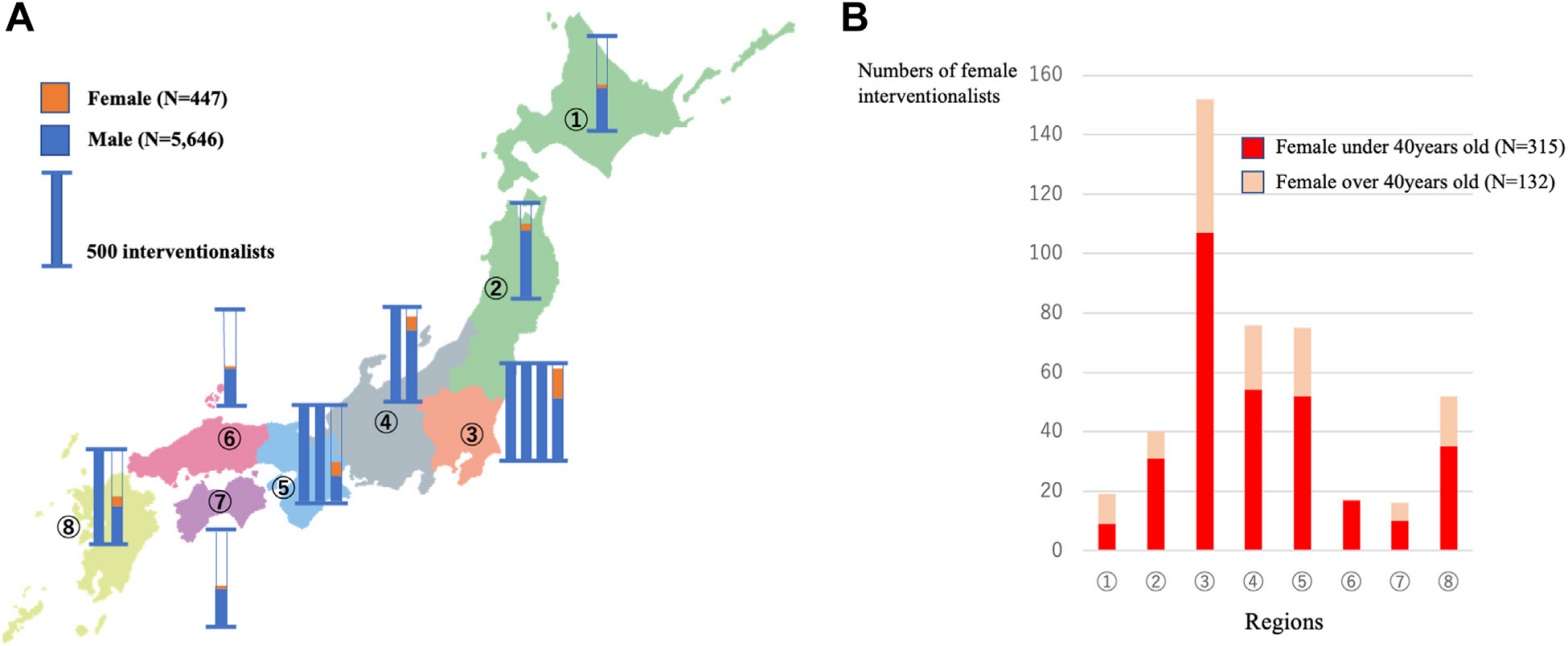
Distribution of Female Interventionalists Across Japan (A) The map graph illustrates the distribution of operators across 8 regions, with each bar on the graph representing 500 operators. The blue color represents male, and orange represents female operators. The regions are identified by numbers as follows: ①Hokkaido, ②Tohoku, ③Kanto, ④Chubu, ⑤Kinki, ⑥Chugoku, ⑦Shikoku, and ⑧Kyushu. (B) The red color demonstrates female operators younger than 40 years of age and the pink demonstrates those older than 40 years of age. The numbers correspond to the regions as illustrated in A.

Figure 2A presents the geographic distribution of interventionalists across Japan in 2021. The graph on the map indicates the number of operators in each of the 8 regions. The proportion of blue represents male and orange represents female interventionalists. The density of women is higher in urban areas and more than 70% (n = 315) of the 447 women are younger than 40 (Figure 2B). In addition, of the 447 women, only 41 are board-certified interventionalists (9.2%), whereas 1,595 men (28.3%) are board-certified.

Patients’ clinical characteristics are shown in Table 1. Female patients comprised 23.5%. Female doctors tended to treat more women (24.2% vs 23.4%; *P* = 0.02). Patients on dialysis and had a history of PCI, coronary artery bypass grafting, or heart failure were more likely to be treated by men. Compared with procedures performed by male interventionalists, women performed a higher proportion of acute coronary syndromes (ACS) (40.1% vs 37.7%; *P* = 0.004), especially ST-segment elevation myocardial infarction (STEMI) (20.2% vs 17.7%; *P* = 0.001). The rate of PCIs performed during the weekends was similar for both.

**Table 1:** Patient Clinical, Angiographic, and Procedural Characteristics

	Total Operators (N = 669,379)	Male Operators (n = 634,168)	Female Operators (n = 35,211)	*P* Value
Age, y	71.2 ± 11.3	71.2 ± 11.3	71.0 ± 11.4	0.24
Men	512,259 (76.5)	485,564 (76.6)	26,695 (75.8)	0.02
Hypertension	507,544 (75.8)	480,986 (75.8)	26,558 (75.4)	0.50
Diabetes	302,217 (45.1)	286,257 (45.1)	15,960 (45.3)	0.86
Hyperlipidemia	449,715 (67.2)	426,085 (67.2)	23,630 (67.1)	0.59
Smoking	201,121 (30.0)	189,454 (29.9)	11,667 (33.1)	0.02
CKD	155,791 (23.3)	147,662 (23.3)	8,129 (23.1)	0.24
Dialysis	48,715 (7.3)	46,310 (7.3)	2,405 (6.8)	0.002
COPD	19,808 (3.0)	18,706 (3.0)	1,102 (3.1)	0.26
Peripheral artery disease	54,365 (8.1)	51,473 (8.1)	2,892 (8.2)	0.06
History of PCI	300,750 (45.6)	285,258 (45.7)	15,492 (44.5)	0.048
History of CABG	22,155 (3.4)	21,153 (3.4)	1,002 (2.9)	<0.001
History of heart failure	104,768 (16.0)	99,565 (16.0)	5,203 (15.1)	0.04
History of AMI	149,688 (22.8)	141,873 (22.8)	7,815 (22.6)	0.36
ACS	250,921 (37.8)	236,917 (37.7)	14,004 (40.1)	0.004
STEMI	118,597 (17.9)	111,562 (17.7)	7,035 (20.2)	0.001
Non-STEMI	42,828 (6.5)	40,739 (6.5)	2,089 (6.0)	0.58
Unstable angina	89,496 (13.5)	84,616 (13.5)	4,880 (14.0)	0.18
Cardiac arrest within 24 h	13,552 (2.1)	12,814 (2.1)	738 (2.1)	0.67
Acute heart failure within 24 h	28,869 (4.4)	27,320 (4.4)	1,549 (4.5)	0.18
Cardiac shock within 24 h	23,926 (3.7)	22,678 (3.6)	1,248 (3.6)	0.22
Hemoglobin, g/dL	13.2 ± 2.1	13.2 ± 2.1	13.3 ± 2.1	0.03
Antiplatelet therapy	623,018 (93.1)	590,488 (93.1)	32,530 (92.4)	<0.001
Access site				<0.001
TFI	135,272 (20.2)	128,679 (20.3)	6,593 (18.7)	
TRI	498,302 (74.4)	471,243 (74.3)	27,059 (76.8)	
Diseased vessels				
1 vessel	428,118 (64.0)	405,154 (63.9)	22,964 (65.2)	0.10
2 vessels	163,141 (24.4)	154,747 (24.4)	8,394 (23.8)	0.33
3 vessels	75,821 (11.3)	72,024 (11.4)	3,797 (10.8)	0.15
Artery				
LAD	339,432 (50.7)	321,608 (50.7)	17,824 (50.6)	0.63
LCX	152,807 (22.8)	144,894 (22.8)	7,913 (22.5)	0.34
RCA	213,456 (31.9)	202,136 (31.9)	11,320 (32.1)	0.90
Left main trunk	39,120 (5.8)	37,401 (5.9)	1,719 (4.9)	<0.001
Saphenous vein graft	2,012 (0.3)	1,930 (0.3)	82 (0.2)	0.051
Device				
Intra-aortic balloon pump	25,969 (86.0)	24,676 (86.0)	1,293 (86.7)	0.39
Extracorporeal membrane oxygenation	6,297 (20.9)	5,993 (20.9)	304 (20.4)	0.64
Impella	1,947 (6.5)	1,892 (6.6)	55 (3.7)	0.04
Drug-eluting stent	548,805 (82.0)	519,617 (81.9)	29,188 (82.9)	0.07
Rotablator	32,512 (4.9)	31,281 (4.9)	1,231 (3.5)	<0.001
Door-to-balloon time, min	83.5 ± 55.8	83.5 ± 55.9	82.7 ± 54.8	0.94
Fluoroscopy time, min	30.7 ± 25.5	30.8 ± 25.7	29.4 ± 22.3	0.08
Contrast use, mL	128 ± 67.2	129 ± 67.2	126 ± 67.2	0.52
PCI during weekends	55,367 (8.3)	52,506 (8.3)	2,861 (8.1)	0.55

Values are mean ± SD or as n (%).

ACS = acute coronary syndrome(s); AMI = acute myocardial infarction; CABG = coronary artery bypass grafting; CKD = chronic kidney disease; COPD = chronic obstructive pulmonary disease; LAD = left anterior descending artery; LCX = left circumflex artery; PCI = percutaneous coronary intervention; RCA = right coronary artery; STEMI = ST-segment elevation myocardial infarction; TFI = transfemoral intervention; TRI = transradial intervention.

The comparison of angiographic and procedural characteristics between male and female operators is also found in Table 1. Women performed PCI using radial access more frequently (76.8% vs 74.3%; *P* < 0.001). During the procedure, fluoroscopy times were shorter, and less contrast volume was used by female doctors, although there were no significant differences. Male doctors were more likely to perform PCIs for left main disease (4.9% vs 5.9%; *P* < 0.001). The use of a mechanical support device (Impella, Abiomed) or rotational atherectomy (Rotablator, Boston Scientific) was higher among male doctors (3.5% vs 4.9%; *P* < 0.001).

For the primary endpoint, there was no significant difference in in-hospital mortality between male and female interventionalists (1.7% vs 1.8%; *P* = 0.052) (Table 2). Regarding the secondary endpoint, the success rate of PCI by female interventionalists was higher compared with their male interventionalists (97.8% vs 97.2%; *P* < 0.001) (Table 2). In-hospital mortality of STEMI treated by women was lower (4.9% vs 6.0%; *P* < 0.001) (Table 3), whereas the success rate of PCI for STEMI was similar between male and female operators. Concerning the PCI for stable angina, the rate of successful PCI was higher in women (98.3% vs 97.7%; *P* = 0.002) (Table 3). After adjustment, there were no significant differences in in-hospital mortality (adjusted OR [aOR]: 0.896; 95% CI: 0.780-1.03; *P* = 0.12), but the rate of unsuccessful PCI (aOR: 0.806; 95% CI: 0.718-0.904; *P* < 0.001) (Table 4) was lower for female interventionalists.

**Table 2:** In-Hospital Outcomes of All PCIs

	All Operators (N = 669,379)	Male Operators (n = 634,168)	Female Operators (n = 35,211)	OR (95% CI)	*P* Value
In-hospital mortality	12,304 (1.8)	11,699 (1.8)	605 (1.7)	0.880 (0.774-1.00)	0.052
Success of PCI	651,060 (97.3)	616,630 (97.2)	34,430 (97.8)	1.289 (1.149-1.447)	<0.001
Periprocedural myocardial infarction	3,977 (0.6)	3,825 (0.6)	152 (0.4)	0.868 (0.665-1.13)	0.30
Cardiac tamponade	1,015 (0.2)	972 (0.2)	43 (0.1)	0.791 (0.553-1.13)	0.20
Cardiogenic shock	6,250 (0.9)	5,941 (0.9)	309 (0.9)	0.841 (0.687-1.03)	0.09
In-hospital stent thrombosis	995 (0.1)	931 (0.1)	64 (0.2)	1.21 (0.851-1.71)	0.29
Emergent surgery	590 (0.1)	563 (0.1)	27 (0.1)	0.878 (0.504-1.53)	0.64
Bleeding	2,654 (0.4)	2,519 (0.4)	135 (0.4)	1.01 (0.782-1.30)	0.95
Access site bleeding with blood transfusion	1,445 (0.2)	1,369 (0.2)	76 (0.2)	1.07 (0.780-1.46)	0.68
Nonaccess site bleeding with blood transfusion	1,261 (0.2)	1,200 (0.2)	61 (0.2)	0.889 (0.607-1.30)	0.55

Values are n (%).

PCI = percutaneous coronary intervention.

**Table 3:** In-Hospital Outcomes Based on Clinical and Lesion Presentations

	Male Operators (n = 5,646)	Female Operators (n = 447)	Odds Ratio (95% CI)	*P* Value
In-hospital mortality				
STEMI	6,730/111,562 (6.0)	344/7,035 (4.9)	0.781 (0.681-0.895)	<0.001
Stable angina	481/218,840 (0.2)	30/11,824 (0.3)	1.25 (0.681-2.31)	0.47
3 vessels disease	2,292/66,105 (3.5)	117/3,491 (3.4)	0.960 (0.755-1.22)	0.74
Left main disease	1,656/25,734 (6.4)	75/1,209 (6.2)	0.989 (0.732-1.34)	0.95
Rotablator	466/31,281 (1.5)	22/1,231 (1.8)	1.22 (0.692-2.15)	0.49
Success of PCI				
STEMI	108,761/111,562 (97.5)	6,868/7,035 (97.6)	1.065 (0.877-1.294)	0.53
Stable angina	213,900/218,840 (97.7)	11,620/11,824 (98.3)	1.357 (1.114-1.653)	0.002
3 vessels disease	63,037/66,105 (95.4)	3,355/3,491 (96.1)	1.247 (1.002-1.550)	0.048
Left main disease	24,870/25,734 (96.6)	1,173/1,209 (97.0)	1.000 (0.658-1.517)	1.00
Rotablator	30,527/31,281 (97.6)	1,214/1,231 (98.6)	1.261 (0.704-2.262)	0.44

Values are n/N (%).

Abbreviations as in Table 1.

**Table 4:** Multivariate Analysis on Outcomes by Female Operators

	OR (95% CI)	*P* Value
In-hospital mortality	0.896 (0.780-1.03)	0.12
Unsuccessful percutaneous coronary intervention	0.806 (0.718-0.904)	<0.001
Periprocedural myocardial infarction	0.921 (0.705-1.20)	0.54
Cardiac tamponade	0.786 (0.550-1.12)	0.19
Cardiogenic shock	0.875 (0.712-1.07)	0.20
In-hospital stent thrombosis	1.21 (0.863-1.69)	0.27
Emergent surgery	0.783 (0.453-1.36)	0.38
Bleeding	1.10 (0.854-1.42)	0.46
Access site bleeding with blood transfusion	1.13 (0.827-1.53)	0.45
Nonaccess site bleeding with blood transfusion	0.911 (0.624-1.33)	0.63

## Discussion

Our report highlights the following salient findings (Central Illustration). First, female operators remain uncommon in Japan, comprising only 7.3% (447 of 6,093) of all interventionalists, and performing only 5.3% (n = 35,211) of all PCI procedures over 3 years. Second, female operators treated a higher proportion of patients with ACS, particularly STEMI, than male operators. Third, the in-hospital mortality rate following PCIs was low at 1.7%, and there was no significant difference observed between male and female operators after adjusting for patients' risk factors. However, the rate of unsuccessful PCI was lower in the female operators. To the best of our knowledge, this the largest study assessing the association between interventionalists’ gender with patient outcomes.^7^, ^8^, ^9^

**Central Illustration:**
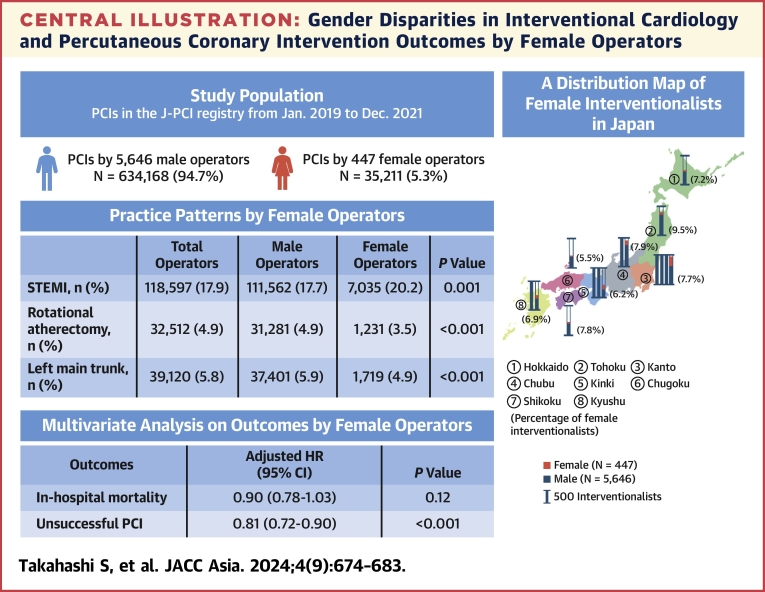
Gender Disparities in Interventional Cardiology and Percutaneous Coronary Intervention Outcomes by Female Operators From January 2019 to December 2021, 447 female operators (7.3%) conducted 5.3% of all PCIs in Japan. Although female interventionalists were relatively few on a global scale, female doctors handled a higher proportion of STEMI patients, and their in-hospital outcomes were found to be comparable to their male counterparts. J-PCI registry = Japanese Percutaneous Coronary Intervention registry; PCI = percutaneous coronary intervention; STEMI = ST-segment elevation myocardial infarction.

### Number of female interventionalists in Japan

The issue of gender disparity in the field of interventional cardiology has been a global concern. Studies from various countries, including the United States,^7^^,^^9^ Australia, New Zealand,^12^ and Europe,^8^^,^^10^^,^^11^ have reported on practice patterns of PCIs performed by women. Data from 2015 in the United States revealed that women accounted for 13.2% (n = 2,908) of general cardiologists and 7.4% (n = 240) of interventionalists.^21^ American women performed only 3% (n = 70,009) of all U.S. PCIs^7^ between 2009 and 2013. In Poland, only 31 female operators (4.1%) performed 2.8% (n = 12,935) of PCIs from 2014 to 2017.^8^ Similarly, in France, women comprised only 3% of interventional cardiologists,^10^ and in Australia and New Zealand, women made up just 4.8% (n = 19) of interventional cardiologists.^12^ Despite the low proportion of female doctors (7.3%) among interventional cardiologists in Japan, our study yielded an unexpected finding that the actual number of female operators, including board members and residents, is relatively high compared with other countries, with a total of 447 (7.3%) female operators (Table 5).

**Table 5:** Proportion of Female Interventionalists in Each Country

Country	Year	All Operators	Female Operators	PCI by All Operators	PCI by Female Operators
United States^21^	2015	3,248	240 (7.4)	NA	NA
Poland^8^	2014-2017	757	31 (4.1)	456,455	12,935 (2.8)
France^10^	2013	1,563	49 (3.0)	NA	NA
Australia and New Zealand^12^	2017-2018	398	19 (4.8)	NA	NA
Japan	2019-2021	6,093	447 (7.3)	669,379	35,211 (5.3)

Values are n (%).

NA = not available; PCI = percutaneous coronary intervention.

### Practice patterns of female operators

Our study found that female interventionalists treated a higher proportion of ACS cases compared with male operators. This finding is consistent with other studies conducted in the United States^7^^,^^9^ and Poland.^8^ Female interventionalists in the United States had a higher proportion of emergency cases such as primary PCI for STEMI or urgent PCI for cardiogenic shock compared with male interventionalists.^7^ Similarly, female operators in Poland treated patients with a lower prevalence of cardiovascular risk factors and mostly single-vessel disease, but ACS was the main indication for treatment (74.7%).^8^ In routine practice, we observed that female interventionalists tended to treat ACS and simpler chronic coronary syndrome patients, whereas male interventionalists were more likely to perform complex PCIs. Men were more likely to be given the opportunity to treat patients with more complex lesions, including left main coronary artery, and had a higher usage of mechanical support devices. Our findings provide further evidence that female interventionalists have a unique practice pattern in Japan.

### In-hospital outcomes of PCI performed by female operators

After conducting a risk-adjusted analysis, we found that the success rate of PCI was higher in female operators, and the clinical outcomes were similar between male and female operators.

In 2013, American College of Cardiology/American Heart Association guidelines^22^ recommended a minimum number of 50 PCI procedures to be performed annually by each operator to achieve better procedural outcomes. Previous reports have consistently shown that the median number of PCIs performed by women per year is considerably lower than that standard,^7^, ^8^, ^9^ which was confirmed by our study. Concerns that low-volume operators would have substantially worse procedural outcomes arose. However, more recent studies have shown that the association between operator volumes and in-hospital mortality has become weaker and operator volume may not be associated with long-term outcomes.^23^^,^^24^ Our analysis revealed that the in-hospital mortality rates for patients treated by women, considered low-volume compared with their male counterparts, were remarkably low at only 1.7%, with no significant difference in mortality rates between male and female operators.

The exact reasons behind the higher success rates of PCI in female operators remain unclear, but there are several potential factors to consider. There are variables not assessed in our dataset, such as the chronic total occlusion lesions that male operators were expected to treat more frequently. In addition, it has been reported that adherence to guidelines by female doctors contributes to better patient outcomes.^9^^,^^13^^,^^24^ Our study did not thoroughly investigate whether PCIs performed by female interventional cardiologists were more aligned with guideline-directed medical therapy.

Despite the lower representation of female interventionalists, their outcomes were found to be fully acceptable. These findings emphasize the importance of increasing the representation of women in interventional cardiology. Our study demonstrates that volume should not be the sole determinant of operator competence. Further research is needed to explore the factors that contribute to these observed differences and to promote gender diversity within the field, while ensuring high-quality care for all patients.

### Future perspectives

Figure 2 presents a map of the distribution of female interventionalists across 8 regions in Japan. Notably, in 3 regions, there are fewer than 20 female interventionalists, whereas there are 7 times more female operators in the Kanto area (Figure 2B). It is also noteworthy that more than 70% of all current female interventionalists in Japan are younger than 40. The actual number of female interventionalists in Japan is not small compared with other developed nations; however, the board-certified female interventionalists are only 0.5% of all interventionalists in this study. Becoming a board-certified interventionalist in Japan requires performing a total of 300 PCIs, which is challenging for many women, as only 13.6% of all female interventionalists perform more than 50 PCIs per year. Young women may face barriers to pursuing this field because of concerns over an uncontrollable or unpredictable lifestyle, long work hours, poor work-life balance, and radiation exposure.^25^ However, our study reveals a positive shift and indicates definitively that women can effectively manage emergencies and odd working hours in the present-day professional landscape. Nonetheless, increasing the number of female interventionalists would create a more diverse workforce, which would be beneficial.^13^^,^^24^^,^^26^ In recent years, the number of women interventionalists has increased slightly in Japan and other countries.^12^ To attract more women to interventional cardiology, it is essential to develop strategies that address the perceived barriers and support career advancement in this field.

### Study limitations

First, although a substantial number of female interventionalists were included in the analysis, not all operators participated in the CVIT registry, which may have introduced selection bias. Second, the COVID-19 pandemic occurred during the study period and may have affected the total number of PCIs performed, although most institutions continued to perform primary PCI for STEMI patients.^27^ Third, our study only collected clinical and procedural data during the hospital stay for the PCI and did not provide further hospital or long-term follow-up data, nor information on medications at discharge, because the J-PCI registry dataset we used for this study did not include long-term outcomes. Fourth, although we do have the age of the cardiologists, which could serve as a proxy for experience, specific data on years of clinical experience are unavailable. In addition, there is a lack of data on cases of chronic total occlusion. These data gaps could potentially affect the interpretation of outcomes. Finally, we did not delve into the factors influencing gender parity in interventional cardiology, which is a crucial topic that warrants further research.

## Conclusions

Although women are still underrepresented in interventional cardiology and perform a lower percentage of PCIs in Japan, our study finds that the practice patterns and outcomes of PCIs performed by female operators are comparable to those of their male colleagues. These findings underscore the importance of promoting gender diversity in interventional cardiology, as it has the potential to enhance patient access to care and ensure equitable outcomes for all patients.

Perspectives **COMPETENCY IN PATIENT CARE AND PROCEDURAL SKILLS:** Gender disparity is an issue in interventional cardiology. Female interventionalists treated a higher percentage of patients with ACS, whereas male doctors were more likely to perform complex PCIs. There was no difference in in-hospital mortality rates, ensuring equal outcomes for patients regardless of the operator’s gender.

**TRANSLATIONAL OUTLOOK:** Research on the factors influencing gender parity in interventional cardiology is needed for promoting gender diversity within the field.

## Funding Support and Author Disclosures

The authors have reported that they have no relationships relevant to the contents of this paper to disclose.
